# Maturity onset diabetes of the young and beyond: the changing face of single-gene diabetes

**DOI:** 10.1093/ejendo/lvaf172

**Published:** 2025-08-16

**Authors:** Thomas W Laver, Kashyap A Patel

**Affiliations:** Department of Clinical and Biomedical Science, University of Exeter, Exeter EX2 5DW, United Kingdom; Department of Clinical and Biomedical Science, University of Exeter, Exeter EX2 5DW, United Kingdom

**Keywords:** maturity onset diabetes of the young, monogenic diabetes, penetrance, polygenic risk

## Abstract

Maturity onset diabetes of the young (MODY) is the most common form of monogenic diabetes. A genetic diagnosis can help tailor treatment. However, it is vital to test the right genes. This review discusses the need to test the correct genes and widen the genes that are included on panels while also discussing examples of genes that should be removed from panels due to insufficient evidence. This is important to ensure the maximum number of patients receive a genetic diagnosis while avoiding misdiagnosis and mistreatment. To maximize the cost-benefit of genetic testing, we need to select the right patients for genetic testing—this review discusses criteria that increase the chance of a patient having a monogenic cause of their diabetes. Reduced penetrance, where some individuals have the pathogenic genotype but do not exhibit the phenotype, is now known to be increasingly common in MODY and other monogenic disorders. This has important implications when a variant is identified in an unaffected individual. This review highlights recent work that polygenic risk may play a role in determining penetrance.

## Graphical Abstract

**Graphical Abstract:**
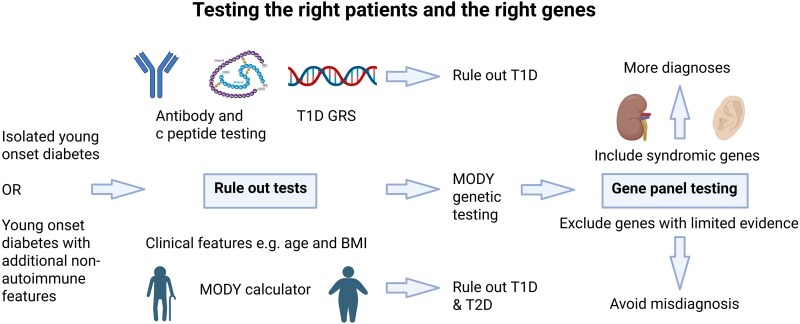

Significance Maturity onset diabetes of the young (MODY) is the most common form of monogenic diabetes. It is at the cutting edge of precision medicine—a genetic diagnosis guides treatment and prognosis. The recent availability of genetic data on large cohorts of cases and controls has allowed novel genes to be identified and previously reported genes to be refuted. It has also provided insight into the variable penetrance seen in MODY and the potential role of common genetic risk factors. This makes MODY an exemplar for understanding the genetic causes of Mendelian disease.

Maturity onset diabetes of the young (MODY) is the most common form of monogenic diabetes, responsible for ∼3% of all diabetes diagnosed under 30 years old.^1^ It is an excellent exemplar for precision medicine as a genetic diagnosis can optimize treatment, for example, patients with HNF1A/HNF4A-MODY respond better to treatment by sulfonylurea tablets than insulin, whereas GCK-MODY does not need any treatment.^2,3^ Maturity onset diabetes of the young is classically thought of as autosomal dominant but that is now known to only capture part of the spectrum of adult-onset monogenic diabetes. This personal point of view will explore recent studies that have made us reconsider which patients to screen, which genes to test, and also which genes not to test.

## Testing the right genes is vital to avoid misdiagnosis

With decreasing cost of DNA sequencing, MODY genetic testing now relies on gene panel testing either by targeted sequencing or virtual panels from exome/genome sequencing. This has changed the focus from only testing the most common MODY subtypes (*HNF1A*, *HNF4A*, and *GCK*) to testing for all subtypes. However, it has also increased the risk of misdiagnosis if genes with poor evidence of pathogenicity are included in panels for monogenic disease. Advancing precision medicine in diabetes and giving each patient the correct diagnosis relies on testing the correct genes. The majority of reported MODY genes were discovered well before large population control datasets, and large MODY cohorts were available. This means some of the genes lacked the crucial scrutiny of their genetic evidence of pathogenicity. Recent research has leveraged 2 of these large population datasets, UK Biobank and gnomAD, to reevaluate the *BLK*, *KLF11*, and *PAX4* genes, over which there were already doubts regarding their pathogenicity.^4^ This showed that many of the published variants were now in population databases at too high frequency to cause MODY and that the cosegregation in the original pedigrees was limited. Most importantly, rare variant aggregation tests were performed and showed that rare variants in these genes are not enriched in a MODY cohort compared with population controls, a crucial piece of genetic evidence that supports a gene being causal for MODY.^5^ These data together suggest that these 3 genes should not be included in gene panels. The growing problem of inclusion of nonpathogenic genes is a wider issue in genetics. This has now been addressed using a consortium of experts supported by the National Institutes of Health under the name of ClinGen^6^; similar efforts are also being undertaken in the United Kingdom under PanelApp.^7^ This work contributed to ClinGen to reclassifying these 3 genes as refuted—not causal for MODY. More recently, a similar approach was used to address the knowledge gap for the genes ClinGen suggested had limited evidence to support or refute the gene–disease relationship. For these genes, using a similar approach, it was shown that dominant rare variants in *APPL1* and *WFS1* are not causative of MODY.^8^ The variant burden tests, however, provided genetic evidence supporting the gene–disease relationships of *NEUROD1* and *PDX1.*^8^

## Redefining genetic heterogeneity in monogenic diabetes

The move to gene panel testing rather than sequential single-gene testing opens up the potential to test a wider range of genes and to take a genotype-first approach—sequencing all diabetes genes in patients rather than sequencing only the specific genes that fit their phenotype. Recent studies have shown that this approach provides a genetic diagnosis for patients who do not have features indicative of their genetic cause. Pathogenic variants in 7 different syndromic diabetes genes accounted for 18% and 19% of all monogenic diabetes cases in 2 suspected monogenic diabetes cohorts not selected for syndromic features.^9,10^ The mitochondrial m.3243A>G variant and variants in *HNF1B* were the most frequent etiologies. Testing genes beyond the traditional dominant MODY genes is of particular importance in populations with high rates of consanguinity. In a study of Turkish pediatric clinics, 41% had recessive causes in contrast to 1.6% in a UK monogenic diabetes pediatric cohort.^11^These studies suggest that genes that typically cause syndromic diabetes should be included on panels in case these patients are referred for MODY genetic testing. This is an easy step to take particularly with the move to virtual panels from exome or genome sequencing; however, accurate variant interpretation is vital. As cases can lack the typical syndromic features, only variants with definite pathogenicity should be reported, and laboratories should refrain from reporting novel variants in this context. Another issue to consider is that reporting of such a diagnosis that may have far more impact on the life of a patient than just diabetes. An early genetic diagnosis before syndromic features have presented can be helpful for the clinician to identify the features early and instigate preventative treatment where possible, but it can also overwhelm the family with completely unexpected findings, making the role of genetic counseling particularly important. These cases may be better managed initially with multidisciplinary teams with expertise in monogenic diabetes.

## Identifying novel genes requires novel approaches

There have been relatively few MODY genes published in the last 10 years. *APPL1* is one example,^12^ but as mentioned earlier, recent data raises questions over that gene–disease relationship. *RFX6* is one example of a recently discovered MODY gene.^13^ Interestingly, it has lower penetrance than previously identified MODY genes, with low penetrance seen even in a clinical setting. Novel MODY genes will perhaps be more like this and less likely the most common causes of MODY such as *HNF1A* where you expect to see large pedigree with high cosegregation. Variants in *RFX6* have lower levels of gastric inhibitory polypeptide hormone; this is in contrast to other forms of MODY or common forms of diabetes but is in keeping with the mouse model data.^13^ The discovery of RFX6-MODY was thus the first human form of diabetes associated with decreased gastric inhibitory polypeptide secretion. An approach that has been successful in identifying novel disease genes is to search for recessive causes of diabetes, particularly in patients with a syndromic presentation. An investigation of children with syndromic diabetes identified recessive loss-of-function variants in *MANF* as a cause of childhood diabetes and a neurodevelopmental disorder.^14^ This builds on an approach that has been highly successful in neonatal diabetes where multiple novel recessive causes have been found by focusing on cases with shared additional pancreatic features. For example, recessive *YIPF5* variants were found by initially searching for the cause of 2 patients with the same extra-pancreatic phenotype before replication studies helped to identify a total of 5 families with a syndrome of neonatal diabetes, epilepsy, and severe microcephaly.^15^ Both *MANF* and *YIPF5* are involved in endoplasmic reticulum stress regulation, and these disease associations have highlighted the importance of this pathway for proper human β-cell function. This reinforces the role of monogenic gene–disease discoveries in identifying important biological pathways as well as directly providing a genetic diagnosis for patients.

## How do we identify who to test?

An important consideration for genetic testing is who should be tested. One option is to test everyone. This is obviously not appropriate given the cost of the test and the rarity of the disease (<3%). Therefore, we do need some strategies to select which individuals with diabetes to perform genetic testing on. This is particularly needed due to the overlapping features of MODY and common type 1 diabetes (T1D) and type 2 diabetes (T2D). There are well-evidenced tools such as MODY probability calculator,^16^ which can be useful in multiple different ethnicities, although some refining is still needed for non-European ethnicities.^17^ The MODY probability calculator uses commonly available clinical features to estimate the probability that the patient has MODY. In addition to using clinical features, there are 2 rule-out tests for MODY, but both have some limitations. The presence of islet autoantibodies rules out MODY if assessed with a well-calibrated assay, and severe insulin deficiency (random c-peptide <200 pmol/L) is also very rare for MODY.^18^ However, islet autoantibodies can be absent in around 10% T1D cases, and this proportion increases with diabetes duration.^19^ C-peptide is less useful near diagnosis due to the honeymoon period in T1D.^20,21^ A recent additional approach has been to investigate common genetic risk factors. It has been demonstrated that common genetic variants that increase risk for T1D can aid in diagnosis by showing that a T1D genetic risk score (GRS) can be used to effectively differentiate monogenic from T1D.^22^ Unlike islet autoantibodies and c-peptide, the T1DGRS is not affected by how close to diabetes diagnosis the sample is taken. Importantly, it can be used in addition to autoantibodies and c-peptide.^23,24^ A further consideration for selection criteria for patients is whether recessive syndromic causes are being screened. In a study of Turkish pediatric clinics, conventional criteria for identifying monogenic diabetes such as parental history, not requiring insulin treatment, and MODY probability calculator score predicted dominant but not recessive cases, which were instead predicted by presence of nonautoimmune extra-pancreatic features.^11^ Although all of these criteria can improve the selection of cases for genetic testing, none will have 100% discrimination. Therefore, the strength of evidence needed before undertaking genetic testing is dependent on individual health care systems. We in the United Kingdom decided to focus on referral criteria that result in around 25% of referrals being positive for monogenic diabetes. However, others may decide to have less strict selection where there is maximum benefit to a genetic diagnosis, for example, in pediatric diabetes. The recent large-scale studies from Swedish and Finnish pediatric clinics that included >86% of all diabetes cases in these countries provided good evidence in this regard. They tested all islet autoantibody–negative cases and found >10% of antibody-negative cases had monogenic diabetes.^25,26^ In healthcare systems where money is not the restrictive factor, there is a case to be made for performing monogenic diabetes genetic testing on all antibody-negative cases. However, this may not work outside pediatric cases due to the rapid rise in T2D. Currently, most countries suggest genetic testing for individuals with diabetes onset <35 years; however, we know that individuals with monogenic diabetes can develop diabetes after this and it is currently unclear if we should expand testing to older individuals A recent study assessed this in >56,000 cases and found 0.44% with monogenic diabetes; however, simple clinical features in combination did not provide enough discrimination to select these individuals.^27^ Therefore, widespread testing in older cases may not be recommended, and case-by-case review is needed.

## Reduced penetrance is now known to be common

One key aspect of the changing face of MODY in recent years is the shift away from the concept that having a monogenic variant guarantees an individual will develop diabetes. Previous studies of penetrance (the probability of individuals with the pathogenic variant developing the disease) often focused on clinically referred families ascertained based on early-onset diabetes, which overestimated the risk. We now know this was an overestimate due to the access to well-phenotyped large population cohorts such as the UK Biobank.^28^ These large cohorts have made it possible to study the penetrance of rare monogenic disorders in clinically unselected cohorts. Mirshahi *et al.*^29^ showed that individuals with HNF1A/4A MODY in population cohorts have 3-5 times less risk of diabetes compared with clinically selected cases. This observation of reduced penetrance in population cohorts has now been seen in multiple diseases^30^ and even for mitochondrial variants.^31^ Interestingly, *GCK* appears to be one of the rare exceptions where variants in a gene retain their consistent effect on phenotype even when identified in a population setting.^29^ This may reflect the pathogenic mechanisms of GCK-MODY whereby GCK acts as a glucose sensor in beta cells, and variants lower the function of the gene, raising the level at which glucose is maintained. The compensation from normal allele that is posttranslationally upregulated by glucose has been proposed as an underlying reason for the consistent phenotype.^32^ These studies demonstrate that caution needs to be exercised when MODY variants are identified outside of a clinical context such as private testing in healthy individuals or as part of research studies. Counseling these cases based on the risk estimates from clinically ascertained cohorts is not appropriate. Further studies in large cohorts are needed to provide an accurate risk estimate for informed discussions.

## Potential role of polygenic risk in the onset of diabetes in MODY

These large-scale studies in clinically unselected cases suggest that there are additional factors in play, which may contributes to development of diabetes in pathogenic variant carriers. However, the conventional view of monogenic disorders such as MODY is that the disease is predominantly due to the pathogenic variant itself with little contribution from other factors such as polygenic risk. Recent research has challenged this view. Variants have been shown to act additively in modifying the penetrance and expressivity of monogenic variants in model organisms.^33^ In humans, polygenic risk has been shown to modify the penetrance of monogenic variants in a range of diseases, including breast cancer and familial hypercholesterolemia.^34,35^Type 2 diabetes genetic risk is an obvious candidate for modifying disease risk in MODY due to shared etiology. If MODY is a solely monogenic disease, the hypothesis is that cases with confirmed monogenic variants will have the same level of T2D polygenic risk as the population. This is similar to what has been shown with T1D genetic risk in monogenic cases.^22^ However, if there is an interaction with polygenic risk, then MODY cases with confirmed monogenic variants will have higher T2D genetic risk than the population. This was recently assessed in a cohort of 1462 HNF-MODY patients, and it was found that the T2D-GRS is greatly raised in MODY cases compared with controls. Interestingly, a similar pattern was seen for insulin secretion, fasting glucose, and fasting insulin, whereas MODY cases had similar body mass index (BMI), lipodystrophy, and T1D GRS to controls (*P* > .05).^36^ It was also possible to estimate that overall common genetic variation contributes around 24% of phenotypic variance for MODY. These data are in keeping with the liability threshold concept whereby the onset of clinical disease is caused by an accumulation of factors including monogenic variants and polygenic variants. Increased T2D genetic risk has also been shown to lower the age at diagnosis of patients with monogenic MODY variants, and even within large multigeneration *HNF1A* MODY families.^37^ These findings reinforce the idea that T2D risk variants increase the risk of diabetes by acting on the same physiological mechanisms affected by monogenic variants in MODY genes, rather than through completely separate genetic pathways. Recognizing the shared genetic pathways between MODY and T2D is important, as it highlights the role that identifying MODY genes may play in uncovering potential T2D drug targets. Finally, we can hypothesize that similar polygenic risk may also potentially explain some cases of individuals with MODY-like diabetes who do not have a monogenic variant in a known MODY gene (hereafter referred to as MODY X). Leech *et al*.^36^ analyzed 298 MODY X cases and showed that these individuals have a T2D-GRS which is higher than T2D cases. They also had higher fasting glucose, waist–hip ratio, BMI, fasting insulin, lipodystrophy GRS compared with controls and similar to or higher than T2D cases. These MODY X cases were diagnosed before the age of 30, had a BMI <30 kg/m^2^ (criteria used to exclude T2D cases), and showed a similar age of diagnosis and BMI to MODY cases with a confirmed genetic diagnosis. In individuals who lack a known pathogenic variant in MODY genes and do not have T1D (either clinically or based on biomarkers), some cases may be explained by an excess polygenic risk for T2D and related traits. This is likely to remain a diagnosis of exclusion, and further studies are needed to better understand the natural history of these cases and to guide appropriate treatment. These data provide the first evidence that a polygenic phenocopy of MODY might exist—where extreme polygenic risk causes a MODY-like phenotype in the absence of a monogenic variant, suggesting that not all MODY X cases are due to an undiscovered monogenic variant. However, it is still possible that these cases may have some additional contribution from intermediate-effect variants, such as the *HNF1A* p.E508K variant^38^ or *HNF4A* p.R114W variant.^39^

## Conclusion

Recent research has changed the landscape of MODY from the simple phenotypic description of early-onset noninsulin-treated diabetes to a far more complex genetically heterogeneous disease where monogenic variation and polygenic risk work together. Discovery of novel disease genes continues to shed light on biological pathways associated with diabetes. Low penetrance in population settings means most MODY variants may not be suitable to be reported as incidental findings, with the exception of variants in *GCK*. The changing face of single-gene diabetes presents opportunities and challenges for researchers and clinicians who must always be asking themselves who to test, what to test, and what not to test.
